# Development and Automation of a Bacterial Biosensor to the Targeting of the Pollutants Toxic Effects by Portable Raman Spectrometer

**DOI:** 10.3390/s22124352

**Published:** 2022-06-08

**Authors:** Oleksandra Bandeliuk, Ali Assaf, Marine Bittel, Marie-Jose Durand, Gérald Thouand

**Affiliations:** 1 Nantes Université, ONIRIS, CNRS, GEPEA, UMR 6144, 85000 La Roche-sur-Yon, France; oleksandra.bandeliuk@univ-nantes.fr (O.B.); ali.assaf1@univ-nantes.fr (A.A.); durand-thouand-mj@univ-nantes.fr (M.-J.D.); 2 Tronico-Tame-Water, 26 Rue du Bocage, 85660 Saint-Philbert-de-Bouaine, France; mbittel@tronico-alcen.com

**Keywords:** biosensor, Raman spectroscopy, toxicity, microorganism, pollutant

## Abstract

Water quality monitoring requires a rapid and sensitive method that can detect multiple hazardous pollutants at trace levels. This study aims to develop a new generation of biosensors using a low-cost fiber-optic Raman device. An automatic measurement system was thus conceived, built and successfully tested with toxic substances of three different types: antibiotics, heavy metals and herbicides. Raman spectroscopy provides a multiparametric view of metabolic responses of biological organisms to these toxic agents through their spectral fingerprints. Spectral analysis identified the most susceptible macromolecules in an *E. coli* model strain, providing a way to determine specific toxic effects in microorganisms. The automation of Raman analysis reduces the number of spectra required per sample and the measurement time: for four samples, time was cut from 3 h to 35 min by using a multi-well sample holder without intervention from an operator. The correct classifications were, respectively, 99%, 82% and 93% for the different concentrations of norfloxacin, while the results were 85%, 93% and 81% for copper and 92%, 90% and 96% for 3,5-dichlorophenol at the three tested concentrations. The work initiated here advances the technology needed to use Raman spectroscopy coupled with bioassays so that together, they can advance field toxicological testing.

## 1. Introduction

Access to safe drinking water and the preservation of existing water resources are highly dependent on the ability to monitor water pollutants and ensure their detection in situ. To meet environmental regulatory objectives, the multitude and diversity of substances to be monitored, often at trace levels, represents a major constraint. This has led to the development of a large number of specific and sensitive analytical methods, a hundred of which are European standards [1,2,3,4,5,6,7]. In addition, complementary methods to assess bioavailability and toxicity are critical for determining the true environmental impact of contaminants [8]. Unlike when using physicochemical methods, it is not necessary to presuppose the nature of the pollutants present in the samples. If the sample is toxic or exerts any effect, a bioassay will respond even if the substance responsible was previously unknown. The design of techniques suitable for toxicity assessment of wastewater along with the implementation of basic ecotoxicity tests dates back to the 1940s [9]. These tests were succeeded by several microbial bioluminescent bioassays and biosensors [10,11,12,13]. A test of this kind forms a central part of the technique that was developed in the present study. For a better assessment of environmental toxicity, several research teams have proposed combining the responses of several microorganisms with single parameters such as bioluminescence, respiration, etc. [4,14,15,16,17]. Thus, covering all metabolic events that may be caused by a toxic phenomenon requires the involvement of as many bioelements and, where appropriate, the multiplication of complex genetic manipulations [18]. This was the case when using a fluorescent library of 1870 transcriptomic reporters from *Escherichia coli* K12-MG1655 to report the ecotoxic status of environmental samples [19]. However, their routine use or integration into a biosensor remains very difficult for field applications.

To overcome these difficulties, the number of examined bioelements can be multiplied. In addition, the number of parameters observed in each of them can be increased, and in this case, there is a move toward a multiparametric approach rather than single parameter measurement. Raman spectroscopy provides a multiparametric tool to help understand the physiological changes induced by toxic substances [20,21,22,23,24,25]. This technology has been widely exploited as an alternative optical method for fast microbial detection and identification over the past two decades [26]. Raman spectra of microorganisms are typically complex, with bands (i.e., peaks) deriving from the thousands (or more) of molecules that make up a sample, each with its own unique Raman signature. In other words, Raman spectra can be understood as molecular fingerprints of the samples under study.

In this context, recent advances in Raman spectroscopy offer new research opportunities by providing a non-destructive approach for monitoring metabolic responses to toxic substances [27]. Work by Bittel et al. [28] on a set of four microbial strains (two bacteria, one yeast and one microalga) and four pollutant models (antibiotics, heavy metals, herbicides and phenol compounds), demonstrated that it is possible to determine the place in the cell where the pollutant acts (DNA, proteins, etc.). A statistical analysis strategy was developed on the basis of independent component analysis (ICA). Advantages of the multiparametric aspect of Raman spectroscopy measurements include the ability to split up the overall signal according to the contribution of different cell components and identify the Raman bands, which are decisive for toxic effect detection with statistical calculations. This enables an operator to identify physiological changes and to characterize the bacterial responses to toxic exposure. The step where ’good’ spectra are selected, designed in a previous study [29], permitted a significant improvement in spectral classification according to toxicant concentration.

Current Raman equipment, although well developed for these purposes in the laboratory, is not suitable for use in the harsher conditions of the field. Indeed, through the years, although new Raman spectroscopy techniques have been developed, most of them rely on large microscope systems. These include using metal-coated glass slides [22,30,31,32], automated imaging microscopy [33], laser tweezers Raman spectroscopy (LTRS) [34,35,36,37], LTRS performed on microorganisms directly in the aqueous suspension [38], using a surface-enhanced Raman scattering (SERS) effect [39,40,41] or filtration [42,43,44,45,46].

All of the spectroscopic techniques need to be optimized in further development, to create an automatic approach of measurement and simplifying the cell preparation step. This will generate large amounts of data (continuous, simultaneous measurements of many different samples). The present study proposes, firstly, a new cell biosensor, considering both the way the cells should be prepared and the tested environment, so as to avoid any physiological shifts. Secondly, the present study proposes a dedicated automatic Raman platform for toxicity measurements on living microorganisms to simplify the procedure and reduce analysis time.

## 2. Materials and Methods

### 2.1. Bacterial Culture

The bacterial strain *Escherichia coli* K12-MG1655 (ATCC 700926; genotype, F-lambda-ilvG-rfb-50 rph-1) was used as a model organism. Bacteria were cultivated according to Bittel et al. [29]. Luria-Bertani (LB) medium was used, which was prepared as follows: 1 L of distilled water was supplemented with 10 g tryptone (Biokar Diagnostics, Allonne, France, ref A1401HA), 5 g yeast extract (Biokar Diagnostics, ref A1202HA) and 5 g NaCl (Carlo Erba Reagents, Milan, Italy, ref 479687). Sterilization was performed by autoclaving at 120 ${}^{\circ }$C for 20 min. Starting from 10 mL overnight precultures, 50 mL cultures were generated with an optical density ($OD_{620nm}$) of = 0.1, with both cultures shaken at 250 rpm at 30 ${}^{\circ }$C (Eppendorf Innova^®^ 42 Benchtop incubator shaker, Eppendorf, France). Growth was monitored over time on bacterial culture diluted 1/10 in LB media measured by a spectrophotometer (SAFAS UVmc2) at 620 nm in disposable cuvettes (Brand GmBH, ref 759015) until it reached $OD_{620nm}$ = 0.4, which corresponds to the middle of the exponential growth phase.

### 2.2. Toxic Substances

Three toxic substances, norfloxacin, copper and 3.5-dichlorophenol, were used to represent three families of pollutants present in the environment (antibiotics, heavy metals and herbicides, respectively). A 250 mg.L${}^{-1}$ solution of antibiotic was prepared by adding 25 mg norfloxacin (Sigma-Aldrich, St. Louis, MI, USA, ref N9890-5G) to 100 mL distilled water. A 2 g.L${}^{-1}$ metal solution was prepared by adding 62.6 mg CuSO${}_{4}\cdot$5H${}_{2}$O (Fisher Scientific, Hampton, NH, USA, ref A778701) to 20 mL distilled water. A 10 g.L${}^{-1}$ herbicide solution was prepared by adding 0.25 g 3.5-dichlorophenol (Sigma-Aldrich, ref D7060-0) to 25 mL distilled water. All concentrated stock solutions were prepared in advance, sterilized by filtration using a 0.22 µm porosity cellulose acetate membrane (Dutsher, Brumath, France, ref 146560) in a UV biological safety cabinet (JOUAN MSC 12, France), and then aliquoted and stored at −20 ${}^{\circ }$C in a light-protected place.

### 2.3. Sample Preparation for Toxicant Exposure

STEP 1. The protocol used was adapted from the work of Bittel et al. [29]. After bacteria reached $OD_{620nm}$ = 0.4, the microorganisms were washed to remove any residues of medium or substance that could affect measurement. Washing was done by centrifugation for 5 min at 4800 g and 4 ${}^{\circ }$C (Awel MF20-R), and the pellet was then resuspended in 20 mL of 10${}^{-2}$ M MgSO${}_{4}$ solution (MgSO${}_{4}\cdot$7H${}_{2}$O Sigma, ref M1880) prepared in ultrapure water and then sterilized by autoclaving. For each toxicity test, four Erlenmeyer flasks with biomass production were cultivated separately and then mixed during the washing phases. The cycle of washing was repeated three times.

STEP 2. After washing, the biomass was placed in different Erlenmeyer flasks containing 50 mL of MgSO${}_{4}$ solution to which the desired toxicant concentrations were added. For each tested pollutant, three concentrations were tested in triplicate. The concentrations were chosen to represent, where possible, subtoxic, toxic and lethal concentrations (Table 1). A control sample without toxicant was included in parallel with each test. Initial optical density was 0.4, and incubation was carried out at 30 ${}^{\circ }$C under constant agitation at 250 rpm for 40 min under the same conditions as during the biomass production phase.

STEP 3. After exposure, the microorganisms were washed twice with MgSO${}_{4}$ solution in the same way as in STEP 1, using centrifugation to remove residues of substances that might bias the measurement. Then, 1 mL of MgSO${}_{4}$ solution was added to the biomass pellet, and the samples were centrifuged in 2 mL Eppendorf tubes for 2 min at 10,000 rpm (MinSpin plus, Eppendorf). All liquid was then separated from the bacterial pellet and, finally, 25 µL of MgSO${}_{4}$ solution was added to each sample to give a final volume of 60 µL.

**Figure 1:**
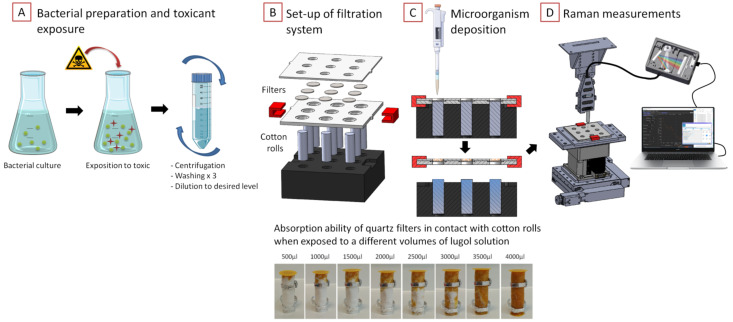
Schema of the toxicity test procedure with the automated spectroscopic system: (**A**) Bacterial preparation and toxicant exposure followed by washing steps to prepare sample for filtration. (**B**) Set-up of multi-well support system for filtration with absorbing cotton rolls and quartz fiber filters. (**C**) Principle of microorganism suspension filtration and liquid absorption by cotton rolls with a function for disconnecting the filters from the wet cotton and fixing them on the motorized 3-axis platform. Demonstration of the ability of quartz fiber filter (diameter 16 mm) to absorb different volumes of Lugol’s solution when in contact with cotton rolls (diameter 12 mm, height 32 mm). (**D**) Automatic spectroscopic measurements with fixed Raman fiber-optic probe head connected to a spectrometer and motorized 3-axis programmed platform simultaneously controlled from a connected computer using a specifically designed API.

STEP 4. This volume of washed cells was then deposited on a quartz fiber filter (Dutscher, grade 293 with a 0.2 µm pore size) using a micropipette. The separated liquid was absorbed using cotton rolls (Teqler, 258 Praxisdient, 12-mm diameter). The filtration of four samples took around 10 min (Figure 1).

### 2.4. Raman Measurements

A confocal Raman microscope (SENTERRA, Bruker Optics, Germany) was used for the measurements of the different filters. This device was equipped with two gratings (400 and 1200 lines/mm), a CCD camera cooled to −60 ${}^{\circ }$C and a BX51 Olympus microscope with multiple objectives (LMPLFLN 100x/0.8 objective, laser spot = 1.12 µm). The analyses were performed at 785 nm, and the laser power was approximately 25 mW on the sample. The spectral resolution was approximately 8 cm${}^{-1}$, and integration time was 30 s with two co-additions. Raman spectra were acquired using OPUS software (Bruker Optics, Ettlingen, Germany).

For field work, we used a portable Raman device (QE Pro-Raman spectrometer, Ocean Optics, Netherlands), hereafter referred to as the ‘Portable Fiber-Optic system’. OmniDriver and SPAM (spectral processing and maths, respectively) libraries were used for the Portable Fiber-Optic system. The spectrometer has low-noise electronics (dynamic range: 85,000:1 and System SNR: 1000:1) with typical back-thinned CCD array miniature spectrometers cooling to −40 ${}^{\circ }$C below ambient air. The spectral resolution was approximately equal to 13 cm${}^{-1}$ in the measured wavelength range (200–4000 cm${}^{-1}$). The fiber-optic probe (InPhotonics RPB785) consists of a permanently aligned combination of two single fibers (105 µm excitation fiber and a 200-µm collection fiber) with filtering and steering micro-optics, in a rugged polyurethane jacket. The stainless-steel probe tip is 38 mm long with a working distance of 7.5 mm. Analyses were performed with laser excitation at 785 nm wavelength and 239 mW power on the control sample run before every experiment with a power-meter (PM100D Thorlabs). The integration time of spectral acquisition was 30 s.

A fiber-optic probe head with a small numeric aperture (NA = 0.22) was selected due to its greater depth of field, thus allowing less precise focusing in the z-direction for a more homogeneous signal from multiple samples.

### 2.5. Design and Fabrication of a Cell Biosensor

The automated spectroscopic system developed in this work is able to measure up to nine biological samples. It consists of a multi-well filtration support system for sample deposition and fixation, a motorized 3-axis platform (Standa, 8-0026) with a fixation base for a sample holder placement, and Raman spectrometer with flexible enhancement and collection arms for easy access to the sample surface. The measuring system is in an enclosed space with an external light-blocking function that allows spectroscopic measurements to be performed in the dark. The system is controlled from a pilot computer with a specially developed application programming interface (API) for simultaneous motorized platform displacement and spectroscopic measurements (Figure 1).

Part 1. Filtration system design

A new optimized filtration method was developed in which the cotton rolls are brought into contact with the filter in order to optimize the absorption of the aqueous medium during filtration (Figure 1B). Nine filters and cotton rolls are fixed in one custom made multi-well support system. The cotton rolls can absorb up to 4 mL of liquid (Figure 1C). The filtration support was designed in the SOLIDWORKS environment and produced from acetal plates using a programmable milling machine (Charly4U); clips to avoid the use of screws for fixing and positioning were produced on a 3D printer (DAGOMA, Neva magis).

Part 2. Set-up of the measurement chamber

The fixation support with the filters is separated from the wet cotton rolls and placed on a custom-made base so they can be positioned on a motorized 3-axis platform (Figure 1B,C). The entire system is secured in a lockable box, coated on the inside with black opaque adhesive film to block out light from the outside and reduce possible reflections of laser radiation on the inside. The Raman probe is fixed in a specially made cage, which is fixed to the ’ceiling’ of the enclosure and has a height adjustment function (Figure 1D).

Part 3. Programming automated spectroscopic measurements

An API was specifically developed to simultaneously operate the motorised 3-axis platform and the Portable Fiber-Optic system. The Libximc cross-platform library was used to control the motorized 3-axis platform.

The API developed makes it possible to select up to nine wells containing samples for measurements and their sequence. The position focusing on the z-axis is first performed manually on the first well. The spectrometer is calibrated with the dark current level and set with the spectroscopic measurement parameters such as the integration time, number of acquisitions and number of runs per well (laser power is set separately with the Oxxius program, LaserBoxx HPE series). Spectroscopic measurements are then run automatically, one spectrum from each sample in sequence, with programmable parameters for the specified number of runs. The API is programmed to measure spectra in seven different spots on the filter (one in the center and six around this point) to homogenize the spectral response from the sample.

Part 4. Data mining for toxicity analysis

Pre-processing

The raw data had a spectral range from 181 to 4045 cm${}^{-1}$. The first pre-processing step was to cut the raw spectra in the defined spectral zone of interest: between 550 and 1780 cm${}^{-1}$ for *E. coli* MG1655. The baseline correction was processed by an elastic concave method (64${}^{\circ }$ and 10 iterations) using OPUS software. Data processing was then performed with MATLAB (version 2019) using the SAISIR package [47]. The spectra were normalized using the probabilistic quotient normalization (PQN) method with respect to the median spectrum of each sample group.

Statistical analysis

The statistics were also performed with MATLAB (version 2019) using the SAISIR package [47]. The statistical analysis strategy was based on independent component analysis (ICA), which was performed using the JADE algorithm [48]. The significance of differences between groups was tested by ANOVA with a statistical significance threshold of *p*-value < 0.05. To quantify the classification results of the spectra, stepwise factorial discriminant analysis (sFDA) procedures were then performed on scores from the ICA selected after the ANOVA analysis. Each discriminant model was calculated using a cross-validation procedure (random selection of 2/3 of scores for the calibration model and 1/3 of scores for the validation test). The final classification rates corresponded to an average of 600 sFDA iterations.

## 3. Results and Discussion

### 3.1. Selection of a Suitable Filter for Bacterial Raman Analysis

**Figure 2:**
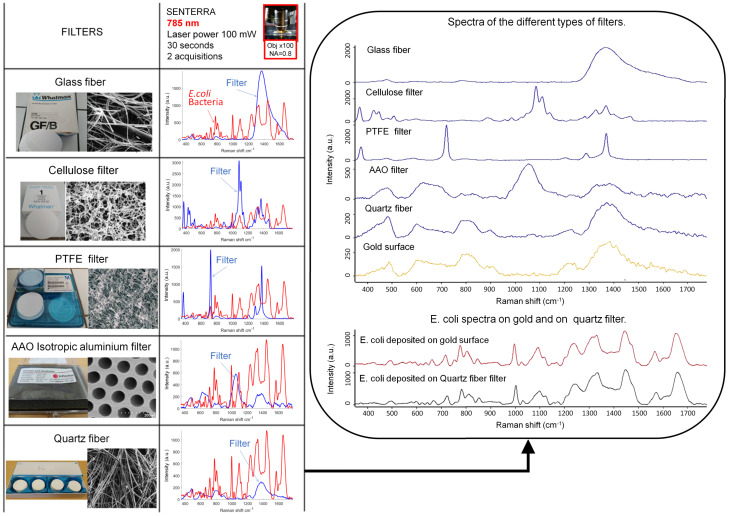
Filter selection based on spectra: (**Left**) Table: Raman signatures of the five tested filters compared with that of *E. coli* MG1655. Blue spectra: filter signatures; red spectra: *E. coli* bacterial signature on a gold surface. All spectra presented were measured at a 785 nm laser excitation wavelength and the same acquisition parameters, using the microscope objective ×100 NA = 0.8. (**Right**) Spectral comparison of the five filters, a clean gold surface and *E. coli* bacteria on a gold surface and on a quartz fiber filter.

Commonly used filter types (glass fiber, cellulose, PTFE, isotropic aluminium and quartz fiber) were tested in order to select the best one for bacterial analysis by Raman spectroscopy. In Figure 2 (left), Raman signatures of the five analyzed filters are shown beside the Raman signal released from *E. coli* deposited on a gold surface. This allows to compare the filter backgrounds, which can overlap with Raman bands of bacteria. Bulk gold is known to be Raman inactive due to its pure face-centred cubic crystal structure [49]. All of the spectra were measured using the same acquisition parameters. Glass, cellulose and PTFE filters have a significant Raman signal at different Raman shifts, making these filters unusable for analyzing bacteria. Isotropic aluminum filters have a weaker Raman response but are very expensive, which is not practical for daily routine applications. Raman signal intensity and band density are smallest for spectra measured on a quartz fiber filter (Figure 2, left). Raman signals recorded from a gold surface and quartz fiber filter are very similar (Figure 2, right). The quartz fiber filter was selected as the best developed Raman biosensor for many reasons: it has a low Raman signal at 785 nm wavelength and is produced with a variety of pore sizes, which ensures an effective retention of bacteria from a filtrated suspension. Moreover, its price is low (0.13 € for 1 cm${}^{2}$), which is important for future field applications. Figure 2 (right) also shows that Raman spectra of bacteria measured on a gold surface and those measured on quartz filter have no visible differences.

### 3.2. Impact of Drying Time on the Quality of Raman Spectra of Bacteria

**Figure 3:**
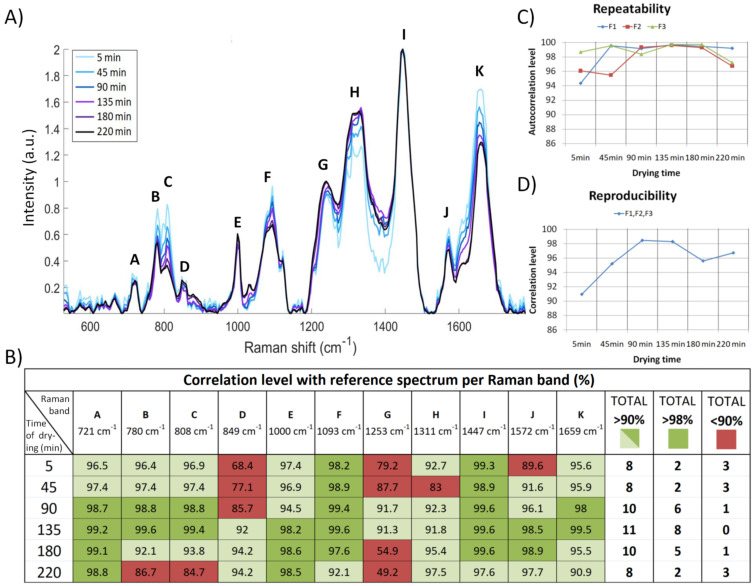
Validation of the best drying time for a bacterial suspension after filtration for subsequent measurement on the semi-automatic system: (**A**) Average of 21 Raman spectra of *E. coli* MG1655 bacteria performed after the same drying time on three quartz fiber filters (seven spectra per filter). (**B**) Average correlation level of each Raman band per drying time with the corresponding Raman band of reference spectrum (*E. coli* deposited on gold surface). (**C**) Repeatability or homogeneity of the measurements represented by the autocorrelation level between seven spectra from each sample for every time interval. (**D**) Reproducibility of measurements represented by the correlation level between all three samples for every time period (21 spectra per time period).

Bacterial cultures were deposited on three quartz filters and measured over six different drying times (5, 45, 90, 135, 180, 220 min). Every spectrum in Figure 3A represents an average of 21 spectra (seven spectra from three samples of filtrated bacteria with identical drying times). Visual inspection of these spectra allows to identify 11 Raman bands impacted by drying time, which were labeled from A to K (Figure 3A,B). It can be seen that the RNA band (808 cm${}^{-1}$, band C) decreases with drying time, which signifies a morphological change in the bacteria. After 220 min, the bacterial RNA band has almost disappeared, which indicates the death of the cell [50]. The best time to extract information from a microorganism about its viability and toxicity response is when a control sample (one not exposed to the toxic substance) has a DNA/RNA ratio of the order of 1 (780 cm${}^{-1}$/808 cm${}^{-1}$), as shown in Figure 3A (bands at positions B and C).

The correlations of the 11 Raman bands with the reference spectrum were calculated to determine at what moment the spectra from filtrated bacteria could be qualified for toxicity evaluation. The reference spectrum is the Raman signal issued from *E. coli* deposited on a gold surface. The average correlation values of each Raman band per drying time with the corresponding Raman band of the reference spectrum are presented in the table of Figure 3B.

All the Raman bands presented were evaluated with >90% or >98% correlation confidence. As can be seen in Figure 3B, during the first hour of measurements, the spectrum of a wet bacterium on a quartz filter is quite noisy, which is reflected by the lower correlation values and, as a result, only two Raman bands have a correlation of >98% with the reference spectrum. The correlation values increase with time and, as can be seen for a drying time of 135 min, eight of the Raman bands have a correlation >98% and all 11 Raman bands have a good correlation, with a reliability >90%. Nevertheless, after 180–220 min of drying, the bacteria begin to degrade, resulting in reductions in the correlations and richness of the spectral information.

To confirm these observations, the repeatability and reproducibility of measurements were calculated to show whether the Raman signal from a sample was stable over time and between samples. The repeatability of measurements is represented by the autocorrelation level between spectra from each sample for every time interval (Figure 3C). The dispersion of values from three samples taken after 5 and 45 min of drying is about 5%, which is very significant in terms of accuracy requirements for toxicity measurements (it should be of the order of 1%). Starting from 90 min of drying time, the dispersion in autocorrelation values between spectra is at an acceptable level of 98%. The reproducibility of measurements illustrates the correlation level between all three samples for every time period (21 spectra per period), which was also calculated (Figure 3D). This value is greater than 98% at 90 and 135 min of drying time. Based on these results, the best time for spectroscopic toxicity measurements on the filtrated bacteria was, therefore, set at between 90 and 135 min after deposition without any impact on the quality of spectra.

### 3.3. Evaluation of the Molecular Targets of Chemical Pollutants in Bacterial Cells

**Figure 4:**
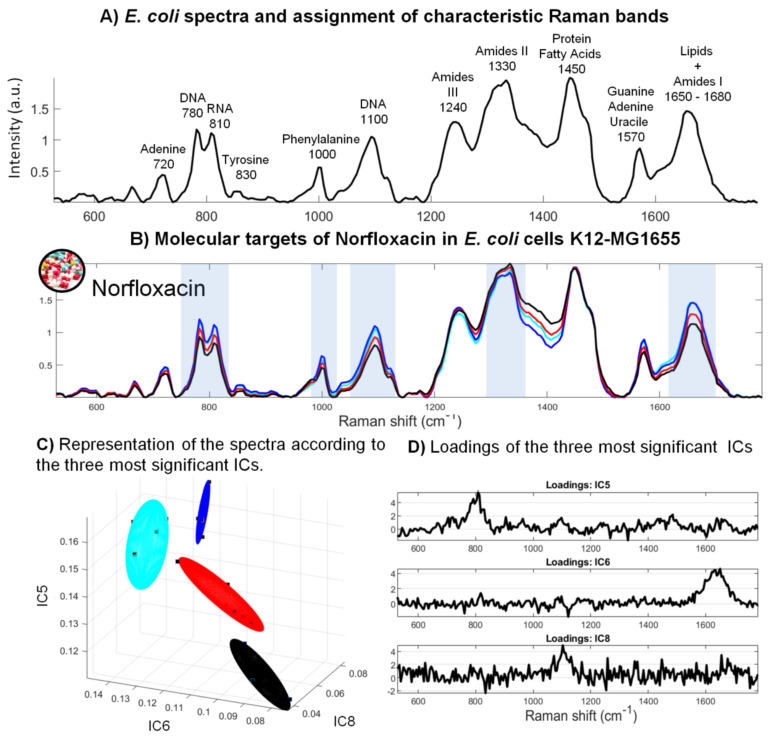

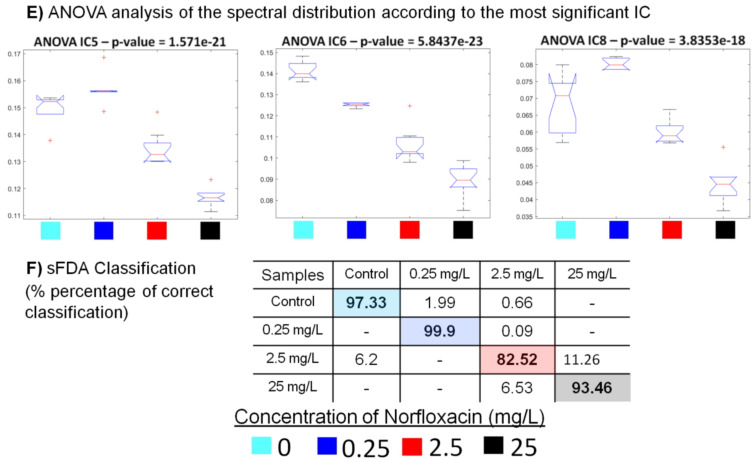
Effects of norfloxacin on the Raman spectra of *E. coli* MG1655: (**A**) Assignment of some characteristic bands in the bacterial spectrum. (**B**) Averages of seven Raman spectra obtained following exposure of the bacteria to different concentrations of norfloxacin. The highlighted bands are those that allow the spectra to be classified according to the different concentrations of toxicant. (**C**) Three-dimensional (3D) representation of the spectral distribution according to the three most significant components from the ICA. (**D**) Loadings of the most significant ICs from the analysis of Raman spectra of *E. coli* MG1655 exposed to norfloxacin. The spectra were decomposed by ICA, and the most significant ICs were then selected. (**E**) ANOVA analysis of the distribution of the spectra according to the most significant component (*p*-value < 0.05). (**F**) Classification results of the sFDA performed after the pre-processing steps of spectrum selection (size of sample: 24 spectra).

The assignment of Raman bands in the reference bacterial spectrum makes it possible to highlight the bands of molecules that may be impacted by the pollutants (Figure 4A). Most biological molecules in a bacterial cell are visible in this spectrum, e.g., adenine (720 cm${}^{-1}$), DNA/RNA (785/850 cm${}^{-1}$), tyrosine (830 cm${}^{-1}$), phenylalanine (1000 cm${}^{-1}$), DNA $-PO_{2}$ phosphate groups (1100 cm${}^{-1}$), group III amides (amides III, 1240 cm${}^{-1}$), group II amides (amides II, 1330 cm${}^{-1}$), proteins and fatty acids (1450 cm${}^{-1}$), guanine adenine and uracil (1570 cm${}^{-1}$), lipids and group I amides (amides I, 1650–1680 cm${}^{-1}$) [28].

#### 3.3.1. Molecular Targets of Norfloxacin in *E. coli* Cells

The analysis of the spectral signature of *E. coli* cells exposed to the different concentrations of norfloxacin shows Raman bands impacted by this toxicant (Figure 4B). The effects concern the DNA and RNA bands at 785 and 850 cm${}^{-1}$, the DNA $-PO_{2}$ phosphate groups at 1070 and 1150 cm${}^{-1}$, amides II (band at 1330 cm${}^{-1}$), amides I and lipids (band at 1650 cm${}^{-1}$). These differences in the molecular fingerprint of the bacteria result from physiological changes provoked by reactions to the antibiotic. These bands, highlighted in Figure 4B, allow the best discrimination of the spectra according to the different concentrations of antibiotic.

The loadings of the three independent components (ICs) show that the variability in the spectra is a function of antibiotic concentration (Figure 4D). It can first be seen that the distribution of the spectra according to these components makes it possible to distinguish the different concentration groups (3D representations, Figure 4C). This selection was made computationally by observing the distribution of spectra for each of the ICs. ICs were selected for which the variability of the inter-group spectra (i.e., as a function of concentrations) was the lowest possible, while maximizing the mean difference with the other groups. The variability of the spectra according to these IC components was also analyzed by ANOVA (Figure 4E). The results show a distribution of groups consistent with a dose–response effect of the substance, which is well underlined by the ANOVA results on the IC6 specific to lipids and amides I (band at 1650–1680 cm${}^{-1}$).

These observed variations are related to known mechanisms of the functioning of this antibiotic [51]. The spectra of bacteria exposed to increasing concentrations of norfloxacin show a decrease in the intensity of the bands corresponding to DNA and RNA (Loading IC5, Figure 4D). Norfloxacin belongs to a family of second-generation quinolones and acts, in particular, by inhibiting DNA gyrase and type IV topoisomerases at the DNA segmentation stage. Inhibition of these enzymes disrupts the DNA segmentation phases, preventing the re-pairing of the strands. This disrupts DNA replication mechanisms, leading to the inhibition of DNA synthesis, which may explain the decrease in the intensity of the corresponding Raman bands. After a period of bacteriostasis, cell death is associated with the appearance of double-strand breaks, causing chromosome fragmentation.

Norfloxacin is known to induce an increase in the production of fatty acids in *E. coli* and a decrease in glycerophospholipid production [51]. Because fatty acids are used by the cell as raw materials for more complex lipids, an increase in fatty acid synthesis combined with a decrease in phospholipid synthesis would be consistent with the activation of cell’s resistance mechanism to compensate for the deterioration of its membrane.

Stepwise factorial discriminant analysis (sFDA) was performed on the scores of independent components from the ICA procedure to assess the level of correct prediction in assigning spectra to a particular group (Figure 4F). Correct classification percentages for the control and the different norfloxacin concentrations (0.25, 2.5 and 25 mg.L${}^{-1}$) were 97, 99, 82 and 93%, respectively. These results, obtained by a portable fiber-optic spectrometer, are comparable to those from a previous study obtained with a benchtop confocal Raman spectrometer [28].

#### 3.3.2. Molecular Targets of Copper in *E. coli* Cells

**Figure 5:**
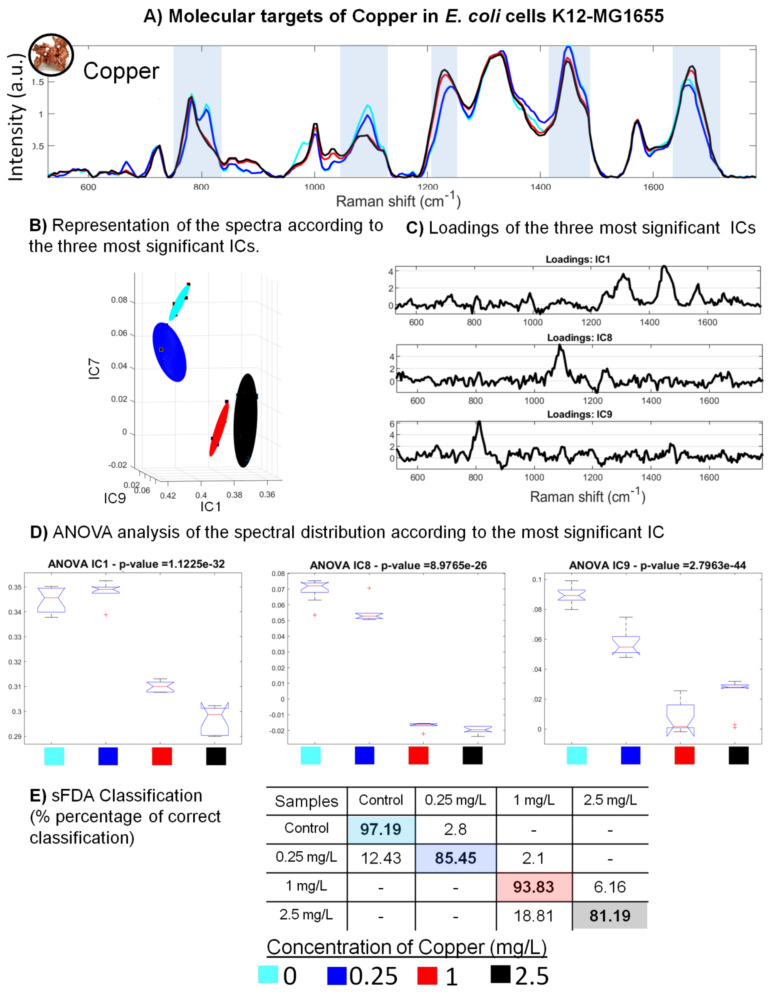
Effects of copper on the Raman spectra of *E. coli* MG1655: (**A**) Averages of seven Raman spectra obtained following exposure of the bacteria to different concentrations of copper. The highlighted bands are those that allow the spectra to be classified according to the different concentrations of toxicant. (**B**) Three-dimensional (3D) representation of the spectral distribution according to the three most significant components from the ICA. (**C**) Loadings of the most significant ICs from the analysis of Raman spectra of *E. coli* MG1655 exposed to copper. The spectra were decomposed by ICA, and the most significant ICs were then selected. (**D**) ANOVA analysis of the distribution of the spectra according to the most significant component (*p*-value < 0.05). (**E**) Classification results of the sFDA performed after the pre-processing steps of spectrum selection (size of sample: 24 spectra).

The analysis of the spectral signature of *E. coli* MG1655 cells exposed to copper shows Raman bands impacted by this toxicant (Figure 5). The most significant spectral changes are in the DNA and RNA bands at 785, 810 cm${}^{-1}$, and bands located between 1050 and 1150 cm${}^{-1}$ associated with the DNA $-PO_{2}$ phosphate groups. The intensity of all these bands decreases with increasing copper concentration (Figure 5B). Nevertheless, two subgroups may be observed: the spectra for low copper concentrations (0 and 0.25 mg.L${}^{-1}$) have the same Raman profiles, in particular for the band at 810 cm${}^{-1}$. For the higher concentrations (1 and 2.5 mg.L${}^{-1}$), the Raman band at 810 cm${}^{-1}$ has disappeared. These very interesting results show that copper heavily impacts nucleic acid bands and causes the death of cells at higher concentrations. Copper toxicity also impacted the bands located between 1200 and 1500 cm${}^{-1}$ associated with amides and proteins. Highlighted by the most discriminating component of the ICA results, these bands are the most significant for characterizing the effects of copper on *E. coli* MG1655 (Figure 5C,D). These ICs were also analyzed by ANOVA (Figure 5E), which confirmed the existence of two sub-groups: one for low concentrations (0 and 0.25 mg.L${}^{-1}$) and another for higher concentrations (1 and 2.5 mg.L${}^{-1}$). The ANOVA done on the IC8 (specific to the DNA $-PO_{2}$ phosphate group band at 1100 cm${}^{-1}$) underlines this result.

It is interesting to note that these results are very similar to those obtained in a previous study showing the impact of arsenic on *E. coli* cells [29]. The corresponding spectral changes may be the result of oxidative phenomena. Indeed, both copper and arsenic induce large numbers of free radicals, which are responsible for the irreversible denaturation of DNA and RNA [52]. In addition, although less significant, other variations also appear in a similar way for these metals. An increased intensity can be observed in the bands located between 1200 and 1400 cm${}^{-1}$, which are associated with amides III, which are characteristic of proteins and lipids (Figure 5B). These changes can be attributed to the denaturation phenomena [53]. Similar spectral changes can be seen for the band corresponding to phenylalanine (1000 cm${}^{-1}$) and the band corresponding to lipids and amides I (between 1650 and 1690 cm${}^{-1}$). In both cases, there is observed not only an increase in the intensity of the latter band with increasing toxicant concentrations but also a slight shift of a few cm${}^{-1}$ to the right. Both copper and arsenic act at the membrane level, notably through chelation mechanisms [52]. The resulting depolarization may explain the slight shift observed in the Raman bands. Copper and arsenic, which have similar toxicity mechanisms, thus seem to induce equivalent variations in the Raman spectra of *E. coli*.

Stepwise factorial discriminant analysis (sFDA) shows good classification scores for the control and the different copper concentrations (0.25, 1 and 2.5 mg.L${}^{-1}$), correct at 97, 85, 93 and 81%, respectively (Figure 5E). Most of the misclassified spectra can be attributed to neighbouring groups. For example, 18.81% of the 2.5 mg.L${}^{-1}$ concentration spectra were attributed to the 1 mg.L${}^{-1}$ concentration group. Similarly, the misclassified spectra of the 0.25 mg.L${}^{-1}$ group were reciprocally attributed to the control (12.43%) and 1 mg.L${}^{-1}$ (2.1%) groups. This classification confirms the visual observations and allows classifying the impact of copper into two modes. For concentrations lower than 0.25 mg.L${}^{-1}$, copper does not impact the bacteria greatly. However, from a concentration of 1 mg.L${}^{-1}$ and above, copper becomes very toxic and causes cell death (as was also observed for the two high concentrations).

#### 3.3.3. Molecular Targets of 3,5-Dichlorophenol in *E. coli* Cells

**Figure 6:**
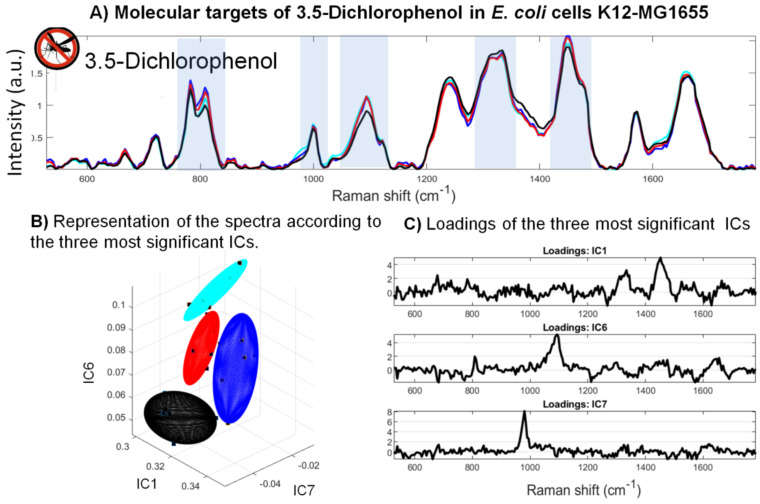

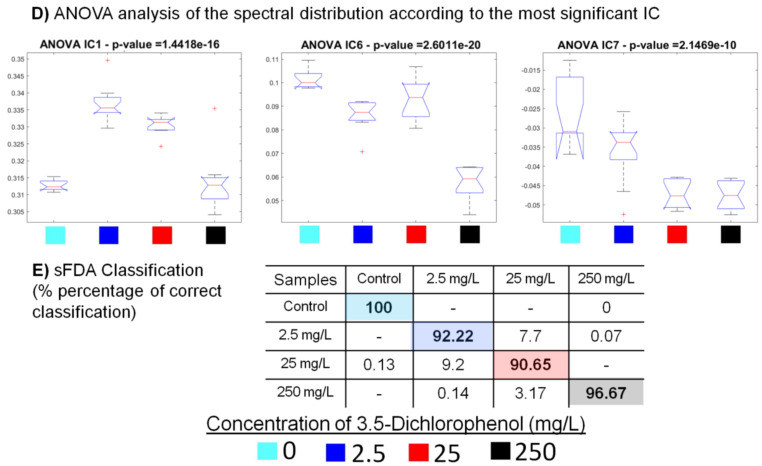
Effects of 3,5-dichlorophenol on the Raman spectra of *E. coli* MG1655: (**A**) Averages of seven Raman spectra obtained following exposure of the bacteria to different concentrations of 3,5-dichlorophenol. The highlighted bands are those that allow the spectra to be classified according to the different concentrations of toxicant. (**B**) Three-dimensional (3D) representation of the spectral distribution according to the three most significant components from the ICA. (**C**) Loadings of the most significant ICs from the analysis of Raman spectra of *E. coli* MG1655 exposed to 3,5-dichlorophenol. The spectra were decomposed by ICA, and the most significant ICs were then selected. (**D**) ANOVA analysis of the distribution of the spectra according to the most significant component (*p*-value < 0.05). (**E**) Classification results of the sFDA performed after the pre-processing steps of spectrum selection (size of sample: 24 spectra).

The analysis of the spectral signature of *E. coli* cells exposed to different concentrations of 3,5-dichlorophenol shows Raman bands impacted by this toxicant (Figure 6). The toxicity signature of this pollutant specifically concerns the bands associated with phenylalanine (1000 cm${}^{-1}$) and the DNA $-PO_{2}$ phosphate groups (1100 cm${}^{-1}$). The intensity of these bands diminishes with increasing concentrations of the toxicant (Figure 6B). A band associated with amides II (1330 cm${}^{-1}$) is slightly higher for the highest concentration of toxicant, although it can be noted that in general, the intensity of this band does not change very much. Highlighted by the most discriminating component of the ICA results, these bands are the most significant for characterizing the effects of 3,5-dichlorophenol on *E. coli* MG1655 (Figure 6C,D). These three IC loadings can be seen to vary, and ANOVA reveals a significant difference between the control and 3,5-dichlorophenol-treated bacteria (*p*-value < 0.05) (Figure 6E).

Phenolic compounds are lipophilic compounds whose toxic effects are mainly driven by their action at the microbial membrane level. Their effect leads to variations in the lipid/protein ratio as well as to the dysfunction of certain membrane proteins. A study by Keweloh et al. [54] on the growth of *E. coli* in the presence or absence of phenols, showed a decrease in the lipid/protein ratio under the effect of the toxicant, in particular due to a decrease in the quantity of phospholipids. Thus, the relative variations of these two bands, recurrently identified in the loadings of the significant ICs, tend to show a relative decrease in the bands associated with lipids compared with those associated with proteins (Figure 6D). Furthermore, the action of phenols would cause an increase in membrane permeability, and the increase in the quantity of membrane proteins would then result in adaptation to limit the leakage of cellular constituents.

Stepwise factorial discriminant analysis (sFDA) demonstrates good classification scores for the control and different 3,5-dichlorophenol concentrations (2.5, 25, and 250 mg.L${}^{-1}$) correct at 100, 92, 90, and 96%, respectively (Figure 6E).

## 4. Conclusions

A newly designed filtration technique for microorganism suspensions coupled to an automated spectroscopic measurement device for up to nine samples opens broad research perspectives for a Raman biosensor development using a Portable Fiber-Optic system. We determined the best parameters for toxicity measurements on filtered bacteria to ensure a favorable repeatability and reproducibility of the tests. These findings demonstrate that it is possible to reduce the number of spectra needed from each sample from 40 to only seven for toxicity evaluation, which reduces the measurement time for four samples from 3 h to 35 min in automatic mode without intervention from an operator. Toxicity tests to validate the new method with the automated spectroscopic system showed good agreement with previous results [28] and the potential for future biosensor development.

Nonetheless, the system developed here remains a device for laboratory use. Furthermore, the systematic analyses of the results are still an integral part of the work required to make a useful interpretation of the information collected. To this end, the development of a model allowing the simultaneous consideration of signatures obtained for all the microorganisms in a given water sample or environment will make it possible to automatically evaluate the information provided by the different cellular components, which can be observed through the combination of their Raman spectra. This future work will require transversal collaboration of different specialities in biology and chemometrics.

## Figures and Tables

**Table 1:** Tested concentrations of the three pollutants in the study.

	Subtoxic	Toxic	Lethal
**Norfloxacin**	0.25 mg.L${}^{-1}$	2.5 mg.L${}^{-1}$	25 mg.L${}^{-1}$
**Copper**	0.25 mg.L${}^{-1}$	1 mg.L${}^{-1}$	2.5 mg.L${}^{-1}$
**3.5-Dichlorophenol**	2.5 mg.L${}^{-1}$	25 mg.L${}^{-1}$	250 mg.L${}^{-1}$

## Data Availability

Not applicable.
